# Optimization of the *Artemisia* Polysaccharide Fermentation Process by *Aspergillus niger*

**DOI:** 10.3389/fnut.2022.842766

**Published:** 2022-03-17

**Authors:** Ali Tao, Xuehua Feng, Yajing Sheng, Zurong Song

**Affiliations:** College of Pharmacy, Anhui Xinhua University, Hefei, China

**Keywords:** *Aspergillus niger*, *Artemisia annua* polysaccharide, optimization, fermentation process, single factor, response surface

## Abstract

In order to investigate the fermentation process of *Artemisia* polysaccharides, this paper showcases an investigation into the effects of fermentation time, fermentation temperature, strain inoculum, *Artemisia annua* addition, and shaker speed on the polysaccharides production of *Artemisia annua*. The yield of *Artemisia* polysaccharides content was determined based on the optimization of single-factor test, and then a response surface test was conducted with temperature, inoculum, and time as response variables and the yield of *Artemisia* polysaccharides as response values. The fermentation process was then optimized and the antioxidant activity of *Artemisia* polysaccharides was monitored using DPPH, ABTS^+^, OH, and total reducing power. The optimum fermentation process was determined by the test to be 5% inoculum of *Aspergillus niger*, temperature 36°C, time 2 d, shaker speed 180 r/min, and 4% addition of *Artemisia annua*, and the extraction of *Artemisia* polysaccharides was up to 17.04% by this condition of fermentation. The polysaccharides from *Artemisia annua* fermented by *Aspergillus Niger* had scavenging effects on DPPH, ABTS, and OH free radicals.

## Introduction

Polysaccharide is a natural biological macromolecular substance with anti-inflammatory (1), anti-tumor, anti-radiation, hypoglycemic, hypolipidemic, and immune-enhancing effects (2). Studies have shown that some plant polysaccharides composed of galacturonic acid and glucuronic acid (3) and can enter the colon and exhibit probiotic properties (4), which may alter specific microbial populations and protect the gastrointestinal tract (5, 6). *Artemisia annua L*. is an annual, strongly scented herb commonly found in Chahar and northern Suiyuan provinces of China at altitudes of 1,000–1,500 m (7). It has been found to have anti-inflammatory, antibacterial, antihypertensive, antimalarial, antioxidant, antiparasitic, immunosuppressive, and collagen-induced arthritic effects (8). It is used clinically for the treatment of respiratory, digestive, skin, endocrine, and febrile diseases (9). The discovery of Artemisinin by Tu Youyou and her team is the first natural drug in China to be internationally recognized (10). It contains sesquiterpene lactones with intracyclic peroxide bonds (11). It is the drug of choice for the treatment of malaria. The main chemical components of *Artemisia annua*, flavonoids, coumarins, sesquiterpenes, and volatile oils, have been extensively reported (12) in large quantities. Although *Artemisia* polysaccharide is one of the active components of *Artemisia*, little research has been carried out on it and its extraction process. In recent years, it has been found that *Artemisia* polysaccharides have an anti-inflammatory effect (13). It can induce apoptosis of cancer cells, improve immunity, and show anti-liver cancer properties (14). Therefore, we can focus on its extraction research. The traditional extraction method of polysaccharides is hot water or boiling water extraction (15), now the extraction methods of polysaccharides from *Artemisia annua* are mostly: ultrasonic leaching method (16), *papain* extraction method (17), and microwave-assisted extraction method (18).

Microbial fermentation can effectively improve the yield of active ingredients of herbal medicines, and some studies have shown that the fermentation of *Aspergillus niger* can produce *cellulase*, decompose plant cell walls, and promote the release of active ingredients (19). In Tao et al. (20) the fermentation of *Astragalus* membranaceus using *Aspergillus niger* resulted in a significant increase in the free berberine content. In Liu et al. (21) the fermentation of *Astragalus* membranaceus using *Aspergillus niger* resulted in a significant increase in the extraction of *Astragalus* polysaccharides after process optimization. In recent years, although microbial fermentation has been widely studied for its low cost and simple operation, it is rarely mentioned for *Artemisia* polysaccharides. Therefore, in this paper, *Aspergillus niger* was selected to ferment *Artemisia annua*, and the process of extracting *Artemisia* polysaccharides was investigated.

## Materials and Methods

### Materials

*Aspergillus niger* (provided by the laboratory of School of Pharmacy, Anhui Xinhua College), dried ground part of *Artemisia annua* (Hefei Chinese herbal market), other reagents were analytically pure.

### Instrumentations

UV2300 Ultraviolet Spectrophotometer (Shanghai Prism Technology Co., Ltd.), Ultra Clean Bench (Haier Co., Ltd.), AL104 Electronic Balance (Shanghai Mettler Toledo Instruments Co., Ltd.), HBM-103 Flow-through Crusher (Hanbo Mechatronics Co., Ltd).

### Test Methods

#### Medium Formulation

Oblique seed medium: the potatoes were peeled and washed, weighed to 200 g and cut into pieces, boiled for 30 min, gauze filtered, then 20 g of glucose was added, along with 15–20 g of agar, which was dissolved and supplemented with enough water to reach 1,000 ml and a natural pH, sterilized at 115°C for 20 min.

Seed medium: a liquid PDA medium without agar.

The bottle of seed medium was shaken: the seed medium was prepared and sealed with eight layers of gauze, sterilized at 121°C for 20 min, cooled to room temperature, and 3~4 pieces of 0.5~1.0 cm^2^ slant strains of surface mycelium (without medium) were inserted into the triangular bottle with liquid volume 50 or 250 ml and incubated 28°C (150 r/min) for 48 h.

Fermentation medium: sucrose 0.15 g, magnesium sulfate 0.021 g, dipotassium hydrogen phosphate 0.1 g, calcium chloride 0.027 g, water in the appropriate amount, sterilized at 121°C for 20 min.

#### Activation and Culture of *Aspergillus niger*

Strain activation: slant seed medium was prepared and sterilized at 121°C for 20 min. The slant medium was made, each sample was slanted into 0.5~1.0 cm^2^ blocks and incubated at 28°C for 72 h. The mycelium evenly grew all over the slant.

Strain culture: *Aspergillus niger* was inoculated in the medium, incubated in the thermostat 30°C until *Aspergillus niger* spores were growing uniformly, then moved to 4°C refrigerator storage spare.

#### Single-Factor Experimental Design

The effects of inoculum, temperature, time, shaker speed, and *Artemisia annua* addition on the yield of *Artemisia* polysaccharides using *Aspergillus niger* and *Artemisia annua* as raw materials were investigated by deep fermentation.

##### Selection of the Optimal Inoculum of Aspergillus niger

The fermentation medium with *Artemisia annua* added at 4% and distilled water 100 ml was inoculated with 3, 4, 5, 6, and 7% *Aspergillus niger*, respectively, and another medium without *Aspergillus niger* inoculation was set as the control group and fermented at 36°C and set to 180 r/min in a shaking bed for 4 d.

##### Selection of the Optimal Temperature

The fermentation medium with *Artemisia annua* was added at 4%, inoculum at 5%, with 100 ml of distilled water, and another medium without *Aspergillus niger* inoculation was set as the control group, and the temperature was set at 32°C, 34°C, 36°C, 38 °C, and 40°C, in order of gradient, and fermented in a shaking bed at 180 r/min for 4 d.

##### Best Time to Visit

The fermentation medium with *Artemisia annua* added at 4%, inoculum 5%, with 100 ml of distilled water, and another medium without *Aspergillus niger* inoculation was taken as the control group, and fermented at 36°C, 180 r/min in a shaking bed for 1 d, 2 d, 3 d, 4 d, 5 d, respectively.

##### Optimal Shaker Speed

The fermentation medium of *Artemisia annua* with an additional amount of 4%, inoculation amount of 5%, with 100 ml of distilled water, and a medium without *Aspergillus niger* was set as the control group, at 36°C, according to the gradient setting of 120 r/min, 150 r/min, 180 r/min, 210 r/min, 240 r/min, shaker fermentation for 4 d.

##### Selection of Optimal Artemisia annua Addition

The fermentation mediums with 5% inoculum and 100 ml of distilled water were added to 2, 3, 4, 5, and 6 %, respectively, and another medium without *Aspergillus niger* was set as the control group and fermented in a shaking bed at 36°C, 180 r/min for 4 d.

#### Response Surface Optimization Tests

Based on the results of the single-factor test, the inoculum amount, temperature, *Artemisia annua* addition, and fermentation time were determined as the main influencing factors with the yield of *Artemisia* polysaccharide as the index. Based on the Box-Behnken (BBD) central combination design principle, the inoculum amount, temperature, *Artemisia annua* addition and fermentation time were selected as independent variables, and the yield of *Artemisia* polysaccharides was set as the response value, and a four-factor, three-level test was set up, and the data were processed by Design-Expert 12.0 software. The experimental factors and levels are shown in Table 1.

**Table 1:** Response surface test factors and levels.

**Factor**	**Level**		
	**−1**	**0**	**1**
A Inoculum amount/%	4	5	6
B Temperature/°C	35	36	37
C Fermentation time/d	1	2	3
D *Artemisia annua* addition/%	3	4	5

#### *Artemisia* Polysaccharide Sample Preparation

*Artemisia annua* was crushed and sifted through 60 mesh to get uniform powder. Four gram of *Artemisia annua* powder was weighed precisely in a 25 ml colorimetric tube, a certain volume of water was added, sonicated, filtered, and water was added to fix the volume to 50 ml to obtain the sample solution of *Artemisia annua* polysaccharides.

#### Determination of *Artemisia* Polysaccharides Content

*Artemisia* polysaccharide is a water-soluble polysaccharide, so the colorimetric method of phenol sulfate was chosen for the determination (22). The content of *Artemisia* polysaccharides was determined by the colorimetric method of phenol sulfate. The basic principle is that the polysaccharide is hydrolyzed by concentrated sulfuric acid to produce monosaccharide, and the glyoxal derivatives generated immediately after dehydration react with phenol under the action of strong acid to produce an orange-yellow substance, and the absorbance value A is linearly correlated with the sugar concentration c at a wavelength of 490 nm and within a certain concentration range.

##### Standard Curve Plotting

After precisely aspirating 0.1mg/ml glucose standard solution of 0.2, 0.4, 0.6, 0.8, 1.0, 1.2, 1.4 ml, respectively into seven test tubes, distilled water was added to make the volume up to 2.0 ml, and 5% phenol was also added at 1.0 ml each and shaken well, before quickly adding concentrated sulfuric acid 5.0 ml, shaking well again, placing the solution in a boiling water bath 15 for min, then in cold water to bring it to room temperature. The absorbance was measured at 490 nm and 2.0 ml of distilled water was used as a control.

##### Sample Content Determination

0.6 ml of the sample solution of *Artemisia annua* was drawn up precisely in a 50 ml volumetric flask, water was added to the mark, 2.0 ml of the diluted solution was taken, and the absorbance was measured as described above Standard Curve Plotting to calculate the polysaccharide yield (23).

Polysaccharide yield (%) = concentration measured by cuvette (μg/ml) × dilution times × volume of solution (ml)/mass of experimental material (g) ×100%.

#### Preparation of *Artemisia annua* Polysaccharides

##### Fermentation Method

Fermentation was carried out by using the optimized process of a single factor experiment. The control group and the experimental group were centrifuged at 10,000 r/min for 10 min, the supernatant was concentrated to 200 ml, added with 4 times the volume of anhydrous ethanol for alcohol precipitation, and then moved to the refrigerator for storage. After 24 h suction filtration, the precipitate is the crude polysaccharide of *Artemisia annua*.

##### Hot Water Method

The hot water method was used to extract the polysaccharides from *Artemisia annua* as the reference extraction method. The final conditions were selected at the time of 4 h, the temperature of 90°C, and the addition of *Artemisia annua* 4%. The treated samples were placed in distilled water for water bath extraction. After extraction, the method of Preparation of *Artemisia annua* Polysaccharides was used to obtain *Artemisia annua* polysaccharide.

#### *Artemisia annua* Polysaccharide Refining

After the crude polysaccharide of *Artemisia annua* was dissolved in water, it was mixed with 4 times the volume of Sevage reagent (chloroform:n-butanol = 4:1), shaken for 30 min, centrifuged at 4,000 r/min for 10 min, before collecting the supernatant and repeating twice (24). After deproteinization, the crude polysaccharide solution was decolorized by AB-8 macroporous adsorption resin method, shaken for 2 h, suction filtered, the filtrate was concentrated to a certain volume, 4 times the volume of anhydrous ethanol was added, then it was moved to a 4°C refrigerator overnight, 10,000 r/min for 10 min, and the pellet was in deionized water. 8,000–14,000 dialysis bags were used for dialysis, the water was changed several times during the dialysis, and the dialysate was freeze-dried after 48 h to obtain the polysaccharide powder of *Artemisia annua* by fermentation method and the polysaccharide powder of *Artemisia annua* by hot water method, which were used for the determination of antioxidant activity.

#### Determination of Antioxidant Activity of *Artemisia* Polysaccharides

##### DPPH^−^ Free Radical Scavenging Rate

Weighed 5 mg of DPPH dissolved with anhydrous ethanol and fixed the volume into a 100ml volumetric flask, stored it away from light for use right after it was ready (25). Added 2.0 ml of DPPH solution to 2.0 ml of Preparation of *Artemisia annua* Polysaccharides. *Artemisia annua* polysaccharides aqueous solution was prepared at different concentrations, shaken well, and let stand for 30 min away from light, and the absorbance was detected at 517 nm. The same volume of deionized water was used as a blank group, the same volume of DPPH was used as background control and the same concentration of ascorbic acid was used as positive control. The scavenging rate of free radicals was calculated as follows.


$$\begin{array}{l} Clearance\;\left(\%\right)=\left(1-\left(A_{\text{sample}}-A_{\text{back}}\right)\right)/A_{\text{empty}} \end{array}$$

##### ABTS^+^ Free Radical Scavenging Rate

Seven millimoles per liter methanolic solution of ABTS was mixed with sodium persulfate and incubated in the dark 16 for h to form ATBS radicals (26). ABTS^+^ was diluted overnight in methanol to obtain an ABTS^+^ solution with an absorbance of 0.70 ± 0.02 measured at 734 nm. The 15.0 ml ABTS^+^ solution prepared by this method was mixed with 2.0 ml of *Artemisia* polysaccharide solution at different concentrations, stirred, and reacted for 10 min at 30°C protected from light, and the absorbance was measured at 734 nm. The same volume of deionized water was used as blank, the same volume of methanol as background control, and the same concentration of ascorbic acid as positive control.


$$\begin{array}{l} Clearance\;\left(\%\right)=\left(1-\left(A_{\text{sample}}-A_{\text{back}}\right)\right)/A_{\text{empty}} \end{array}$$

##### OH^−^ Radical Scavenging Rate

In turn, 1 ml of 9 mol/L ferrous sulfate solution and 2.0 ml of 9 mol/L salicylate alcohol solution was mixed well in a 10 ml colorimetric tube, 1.0 ml of 0.01% hydrogen peroxide solution and 2.0 ml of different concentrations of *Artemisia* polysaccharide aqueous solution were then added (27). The absorbance was measured at 510 nm in a constant temperature water bath at 36°C for 1 h. The same volume of water was used instead of *Artemisia* polysaccharide solution as a blank group, the same volume of water instead of hydrogen peroxide solution as a background control, and the same concentration of ascorbic acid as a positive control, the clearance of hydroxyl groups was calculated as follows.


$$\begin{array}{l} Clearance\;\left(\%\right)=\left(1-\left(A_{\text{sample}}-A_{\text{back}}\right)\right)/A_{\text{empty}} \end{array}$$

##### Total Reducing Power Determination

1.0 ml of *Artemisia* polysaccharide solution was added to 2.5 ml of phosphate buffer solution (pH = 6.6, 0.2 mol/L) and 2.5 ml of 1% potassium ferricyanide solution, mixed well, and reacted in a constant temperature water bath at 50°C for 30 min (28). 2.5 ml of 10% trichloroacetic acid was added, centrifuged at 3,000 r/min for 10 min, The supernatant was taken at 2.5 ml and 2.5 ml of deionized water was added, as well as 0.5 ml of 0.1% ferric chloride solution. The absorbance was measured at 700 nm after 30 min.

### Data Statistics and Analysis

The above experiments were repeated three times and the average value was used as the final test data. Design Expert 12.0 was used for response surface design.

## Results and Analysis

### One-Factor Test

#### Effect of *Aspergillus niger* Inoculum on the Yield of *Artemisia* Polysaccharides

**Figure 1:**
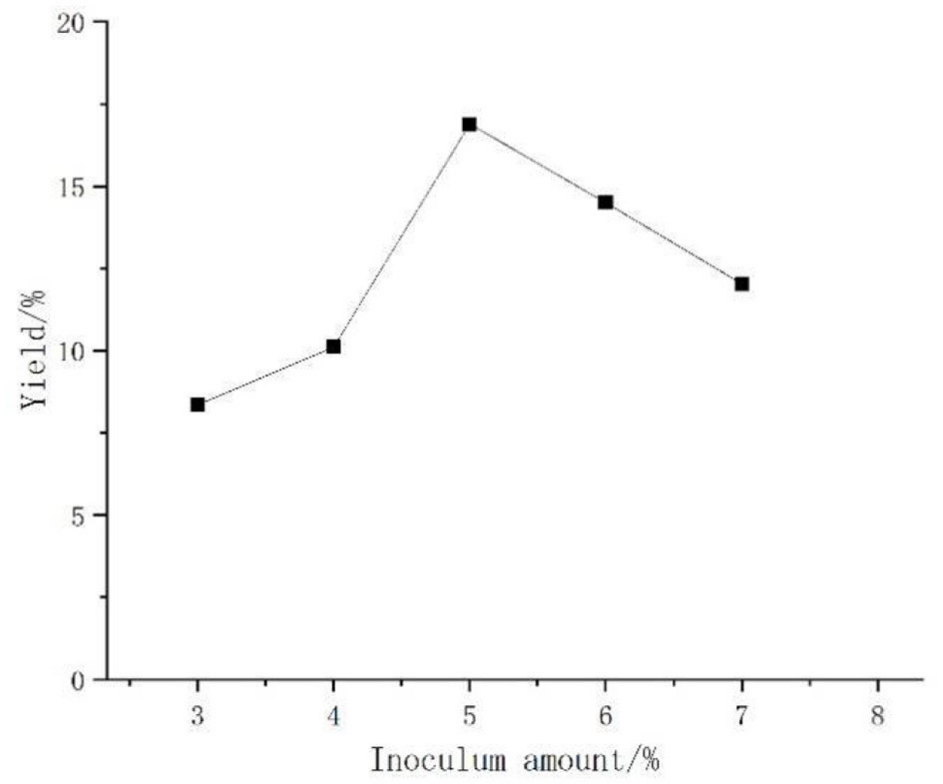
Effect of *Aspergillus niger* inoculum on the yield of *Artemisia* polysaccharides.

*Artemisia* polysaccharide changed pattern as shown in Figure 1, polysaccharide yield with the increase in the inoculum first increased, then decreased in trend, peaking at the inoculum amount of 5% polysaccharide yield, and then tending to decline. The reason may be that due to the fermentation of *Aspergillus niger* in the early stage, polysaccharide production had increased, but because *Aspergillus niger* was aerobic bacteria, the amount of bacteria in the medium was too much, so that the nutrient consumption was too fast, and the polysaccharide yield decreased. Therefore, the inoculum amount of 5% was selected for the follow-up test.

#### Effect of Temperature on the Yield of *Artemisia* Polysaccharides

**Figure 2:**
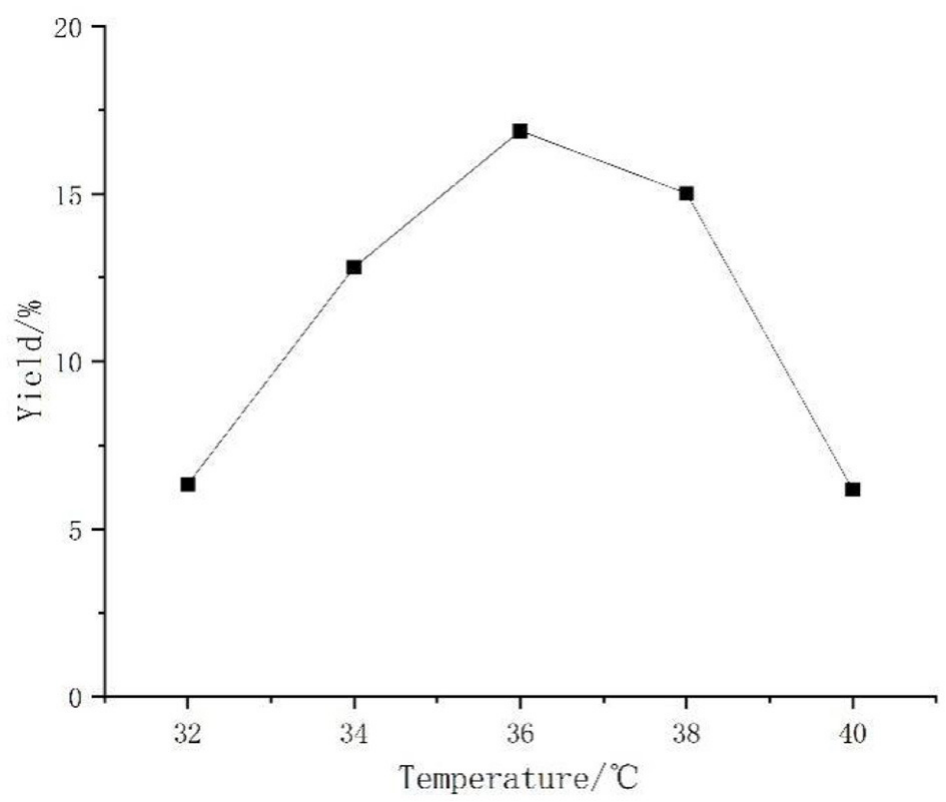
Effect of temperature on the yield of *Artemisia* polysaccharides.

The bacterium needed suitable temperature for reproduction, and when it was too high or low, the enzyme production activity as well as metabolism of the bacterium were affected and the growth of the bacterium was inhibited. In Figure 2, it can be seen that the temperature of the polysaccharide yield had a significant impact. A too high and too low temperature decreased the polysaccharide yield. At more than 38°C, the decline was particularly obvious. The results show that high and low temperatures were not suitable for *Aspergillus niger* fermentation, so 36°C was chosen as the best temperature.

#### Effect of Time on the Yield of *Artemisia* Polysaccharides

**Figure 3:**
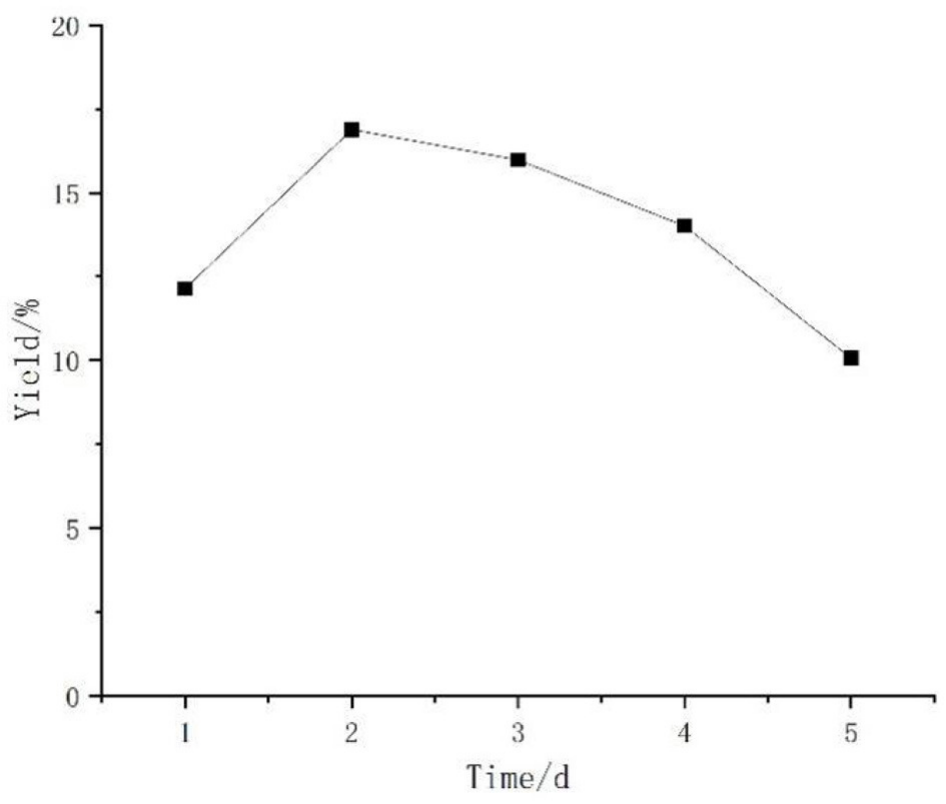
Effect of time on the yield of *Artemisia* polysaccharides.

Figure 3 shows the effect of different fermentation times on the polysaccharide yield. The bacterium grew logarithmically in the early stage of fermentation, accumulated metabolites in the middle and late stage, and then gradually senesced when the growth of the bacterium was stable. The polysaccharide yield in the fermentation broth increased with the increase of fermentation time before 2 d and reached the highest at 2 d. After 2 d, the polysaccharide yield began to decrease, probably due to insufficient nutrient supply and because the consumption of polysaccharide was greater than the production. The results showed that the optimal fermentation time was 2 d.

#### Effect of Shaking Bed Speed on the Yield of *Artemisia* Polysaccharides

**Figure 4:**
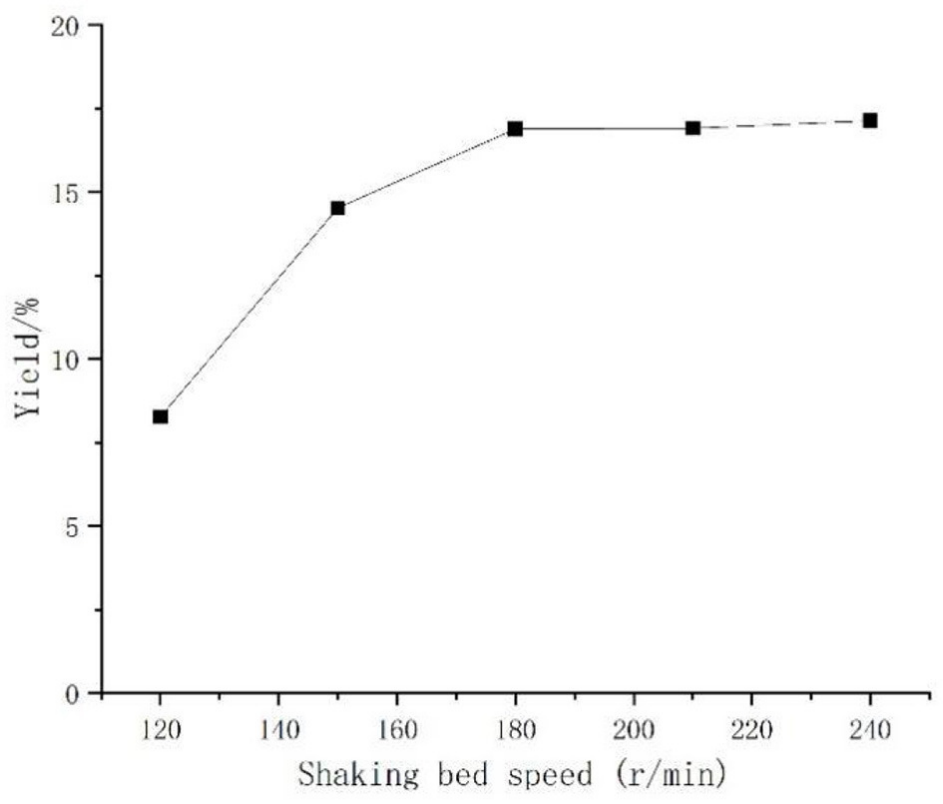
Effect of shaking bed speed on the yield of *Artemisia* polysaccharides.

*Aspergillus niger* is aerobic bacteria, the shaker speed was responsible for regulating the amount of dissolved oxygen. Under the same premise, if the speed was too high or too low, it was not conducive to the growth and reproduction of the bacterium. Observations in Figure 4 found that with the increase of shaking bed speed, the polysaccharide yield rose, but when the speed exceeded 180 r/min, the polysaccharide yield rise was not obvious, considering the actual operation. One hundred eighty revolutions per minute was selected as the best shaking bed speed for *Aspergillus niger* fermentation.

#### Effect of *Artemisia annua* Addition on the Yield of *Artemisia* Polysaccharides

**Figure 5:**
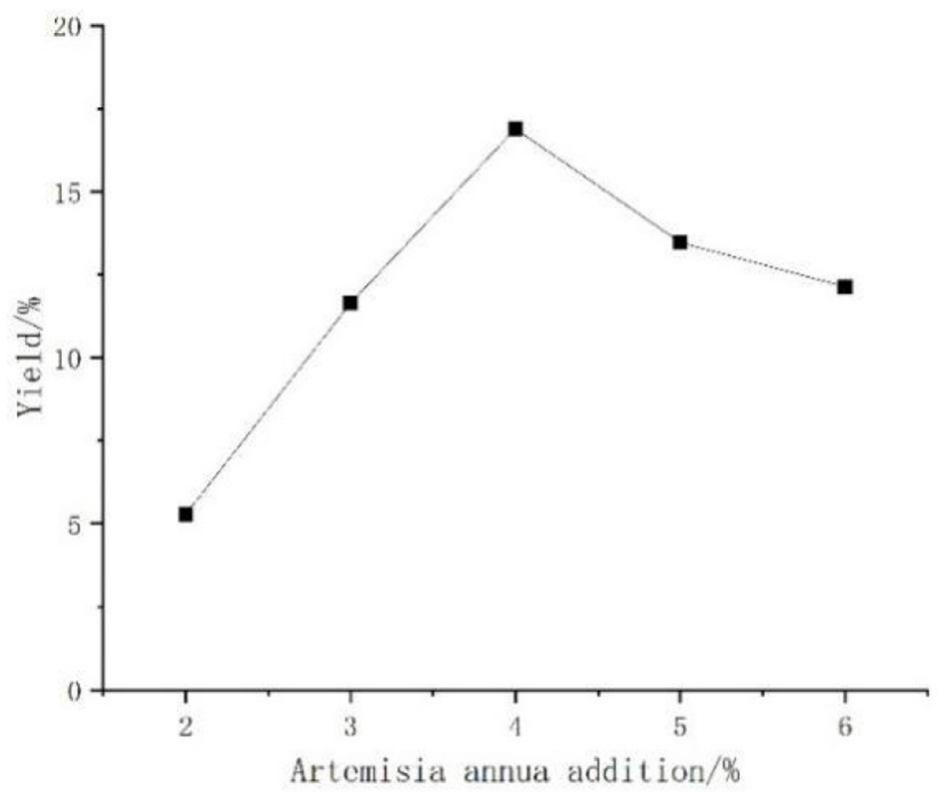
Effect of *Artemisia annua* addition on the yield of *Artemisia* polysaccharides.

The enzymes produced by *Aspergillus niger* fermented *Artemisia*, and when too much *Artemisia* was added, the polysaccharide yield might be affected due to insufficient enzyme production by *Aspergillus niger*. The effect of *Artemisia annua* addition on the yield of polysaccharide was shown in Figure 5, the overall change was relatively gentle, first with the increase of *Artemisia* addition and then slowly increasing, reaching the peak at 4%, after which there was an obvious decline. Therefore, the 4% addition of *Artemisia annua* was the best addition.

### Response Surface Testing

Based on the results of the single-factor test, a Box-Behnken experimental design was conducted with inoculum, temperature, and time as independent variables and *Artemisia* polysaccharide yield as response values, and the results were shown in Table 2.

**Table 2:** Response surface test design and results.

**Test number**	**Inoculum amount/%**	**Temperature/**°**C**	**Time/d**	***Artemisia annua* addition (%)**	**Yield/%**
1	0	1	−1	0	14.72
2	0	−1	0	1	15.40
3	−1	0	0	1	15.86
4	0	0	0	0	16.88
5	0	−1	1	0	15.55
6	0	1	0	1	15.63
7	0	0	1	−1	13.85
8	1	−1	0	0	15.01
9	0	1	0	−1	14.05
10	0	0	0	0	16.68
11	0	0	−1	−1	14.14
12	1	0	1	0	14.64
13	1	0	0	−1	14.10
14	0	1	1	0	14.92
15	−1	1	0	0	15.70
16	0	0	0	0	16.84
17	−1	0	1	0	15.04
18	−1	0	−1	0	14.82
19	0	−1	0	−1	14.56
20	0	−1	−1	0	14.59
21	1	1	0	0	14.86
22	0	0	1	1	16.47
23	−1	0	0	−1	15.49
24	0	0	0	0	17.16
25	1	0	−1	0	14.65
26	1	0	0	1	15.78
27	−1	−1	0	0	15.61
28	0	0	0	0	17.12
29	0	0	−1	1	14.64

Applying Design-Expert 12.0 software to fit the Table 2 to a multiple regression analysis, the regression equation was obtained.


$$\begin{array}{l} Y=16.94-0.29A-0.07B+0.24C+0.63D-0.06AB\\ \;-0.06AC+0.33AD-0.19BC+\;0.19BD+0.53CD-0.78A^{2}\\ \;-0.90B^{2}-1.2C^{2}-0.98D^{2} \end{array}$$

The regression model significance test and anova were performed and the results were shown in Table 3.

**Table 3:** Response surface regression model analysis of variance.

**Source**	**Sum of squares**	**DF**	**Mean square**	**F-value**	***P*-value**	**Significance**
Model	24.73	14	1.77	24.26	<0.0001	Extremely significant
A	1.01	1	1.01	13.86	0.0023	Significant
B	0.0588	1	0.0588	0.8077	0.384	Non-significant
C	0.7057	1	0.7057	9.69	0.0076	Significant
D	4.8	1	4.8	65.95	<0.0001	Extremely significant
AB	0.0144	1	0.0144	0.1978	0.6633	Non-significant
AC	0.0132	1	0.0132	0.1817	0.6764	Non-significant
AD	0.429	1	0.429	5.89	0.0293	Non-significant
BC	0.1444	1	0.1444	1.98	0.1808	Non-significant
BD	0.1369	1	0.1369	1.88	0.1918	Non-significant
CD	1.12	1	1.12	15.44	0.0015	Significant
A^2^	3.91	1	3.91	53.7	<0.0001	Extremely significant
B^2^	5.21	1	5.21	71.59	<0.0001	Extremely significant
C^2^	9.62	1	9.62	132.1	<0.0001	Extremely significant
D^2^	6.17	1	6.17	84.72	<0.0001	Extremely significant
Residual	1.02	14	0.0728			
Lack of fit	0.8572	10	0.0857	2.12	0.2445	non-significant
Pure error	0.1619	4	0.0405			
Cor total	25.74	28				
*R*^2^ = 0.9604	*R*^2^ adj = 0.9208	*R*^2^ pre = 0.7984				
Adeq precision	16.0412					

As seen through the above table, the regression model was highly significant (*P* model <0.0001), the polysaccharide yield misfit term was not significant (*P* = 0.2445 > 0.05), the model had a good fit, and the experimental error was small. The coefficient of determination, *R*^2^ = 0.9604, indicated a good correlation between the measured and predicted values of Y. Over 96.04% of the experimental values could be explained by this equation. The factors affected the degree of *Artemisia* polysaccharide extraction differently, the primary term affected D (<0.0001) > C (0.0076) > A (0.0023) > B (0.384), the interaction AB, AC, AD, BC, BD were not significant (*P* > 0.05), CD was significant (*P* < 0.005), the secondary terms A^2^, B^2^, C^2^, D^2^ were extremely significant (*P* < 0.00001).

#### Response Surface Interaction

**Figure 6:**
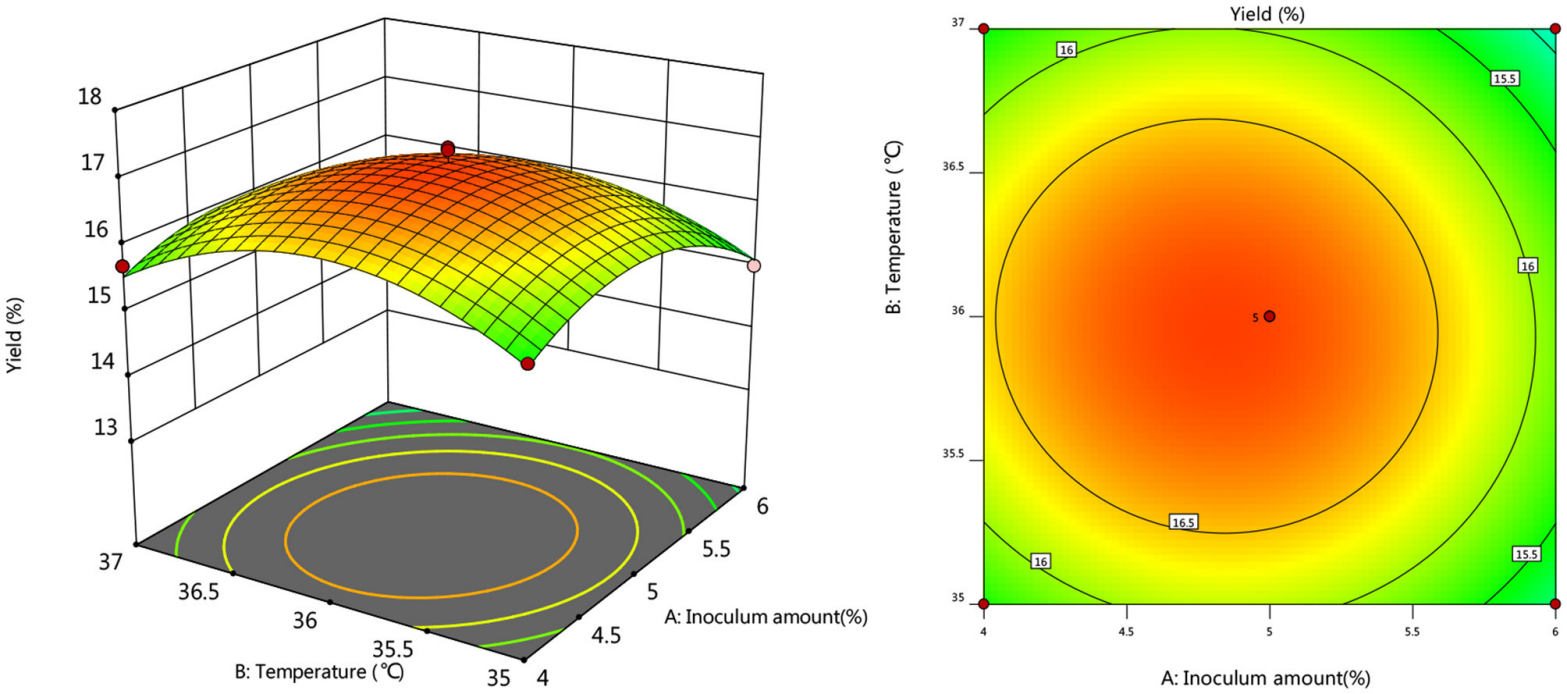
Response plot of the interaction between inoculum and temperature on yield.

**Figure 7:**
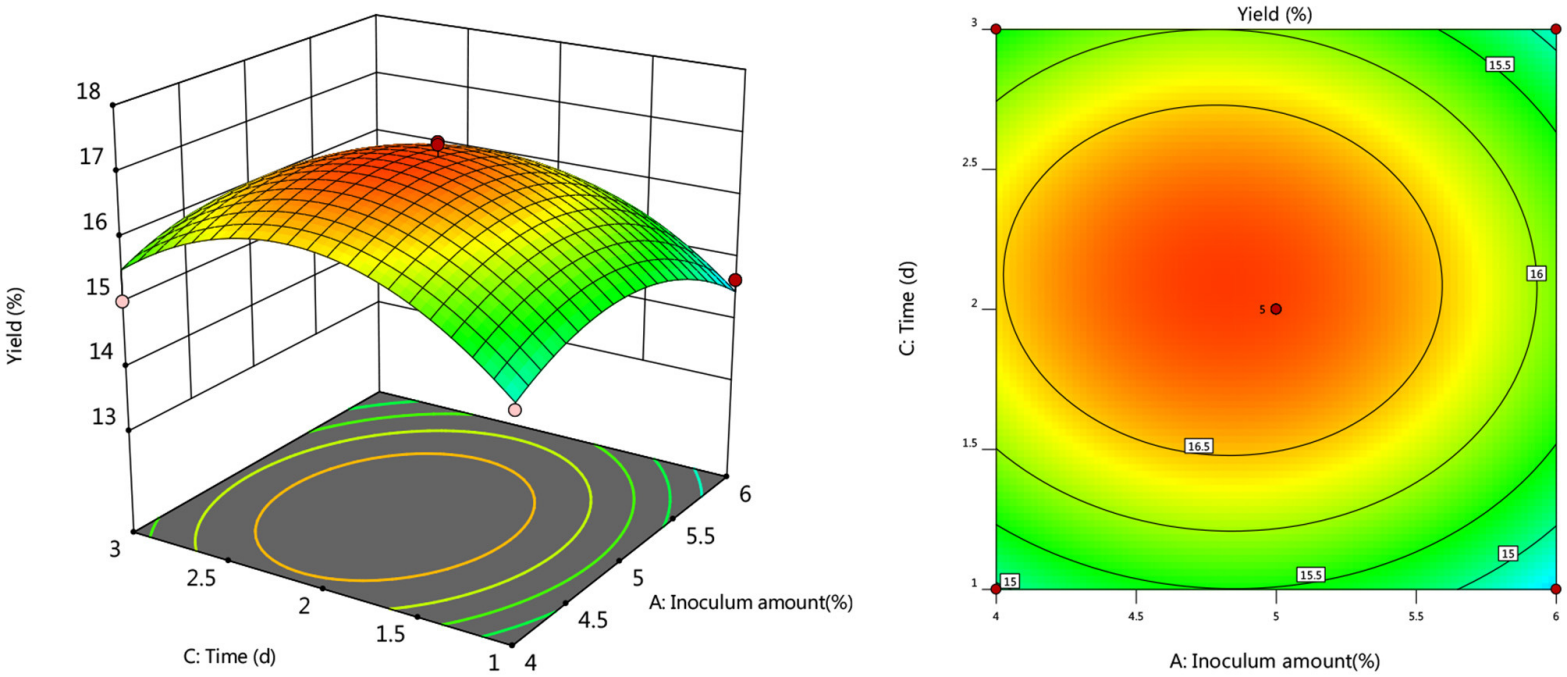
Response plots for the interaction of inoculation volume and time on yield.

**Figure 8:**
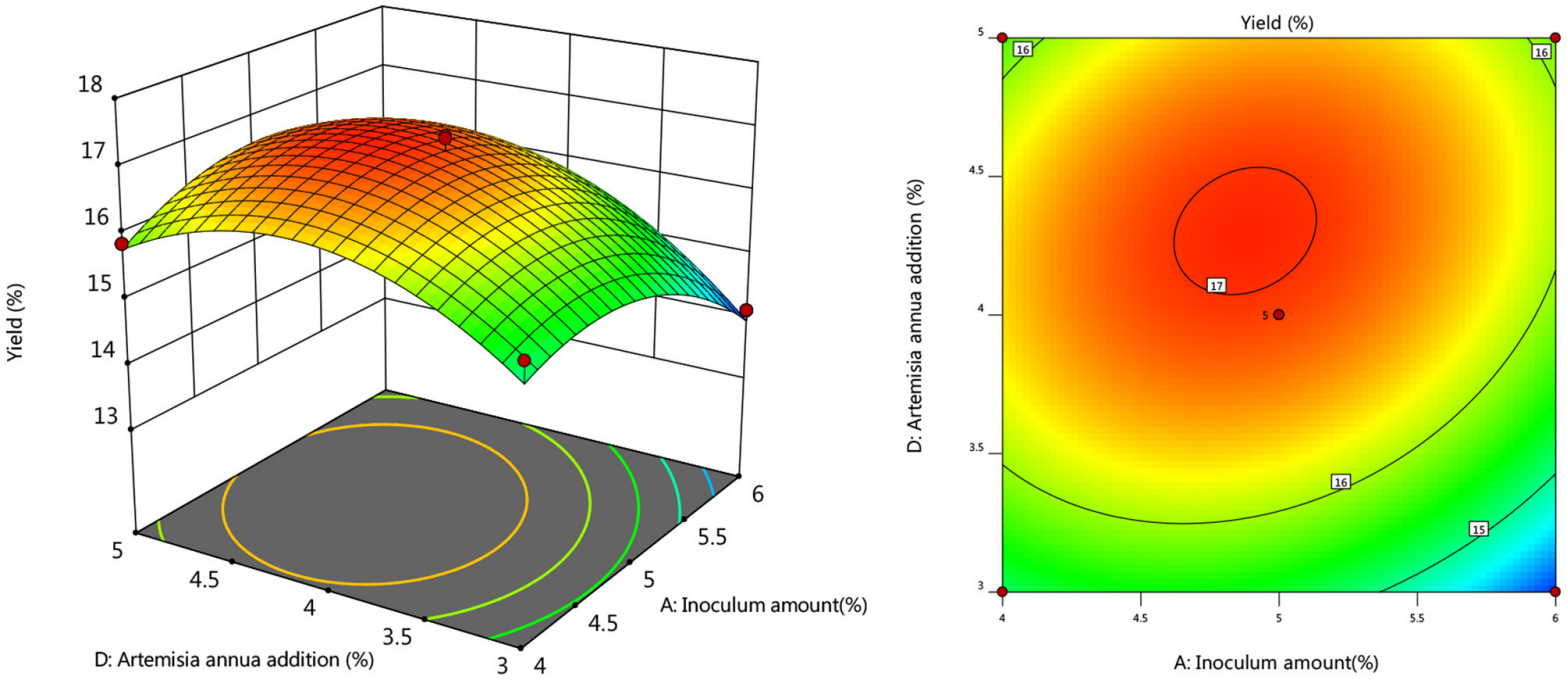
Response plot of the interaction between inoculum and *Artemisia annua* addition on yield.

**Figure 9:**
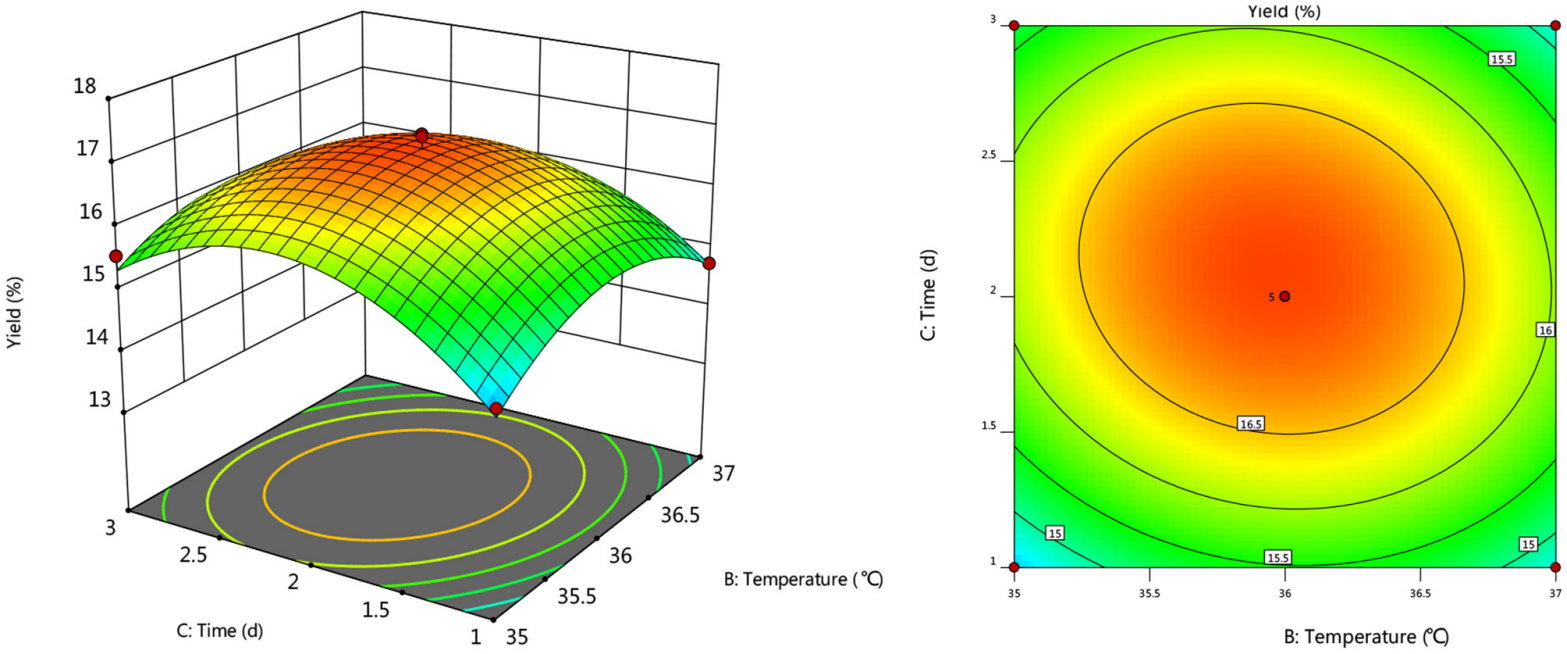
Plot of the response of temperature and time on the interaction of yield.

**Figure 10:**
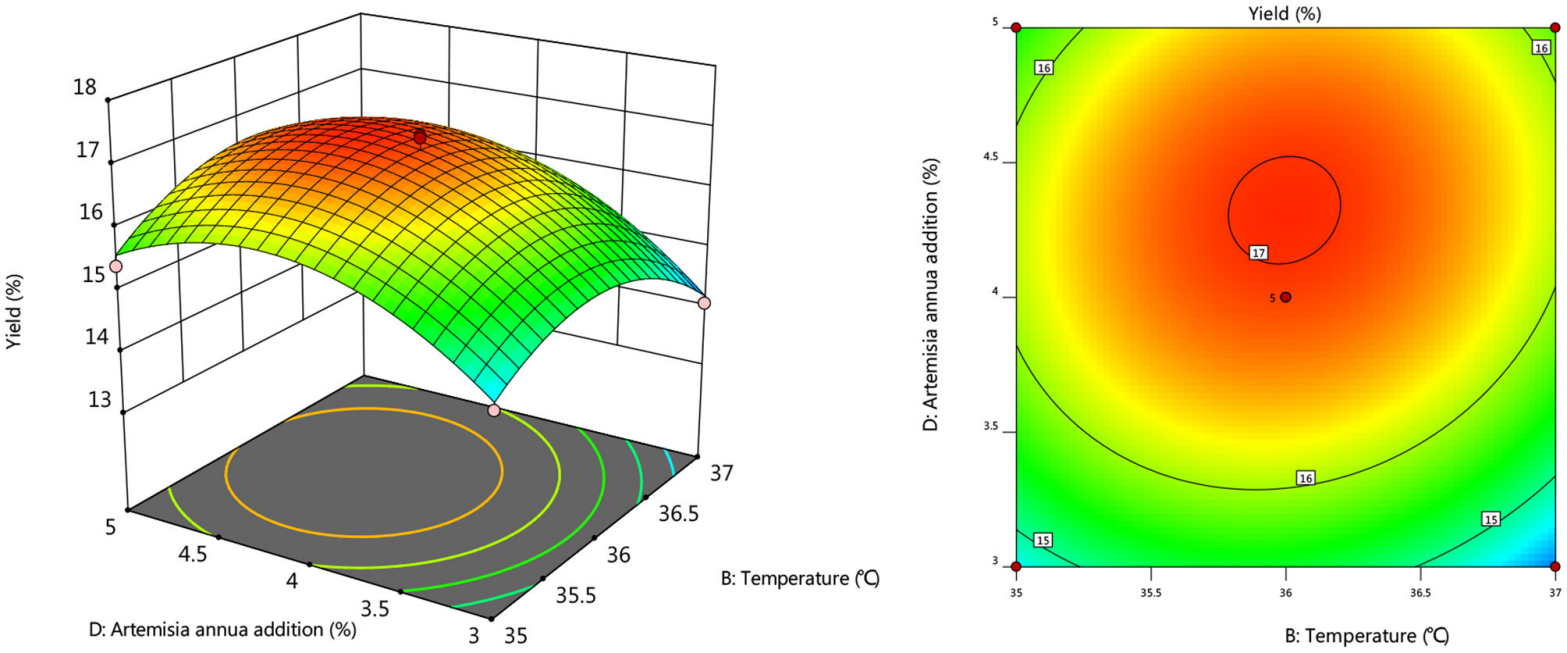
Response plot of the interaction between temperature and *Artemisia annua* addition on yield.

**Figure 11:**
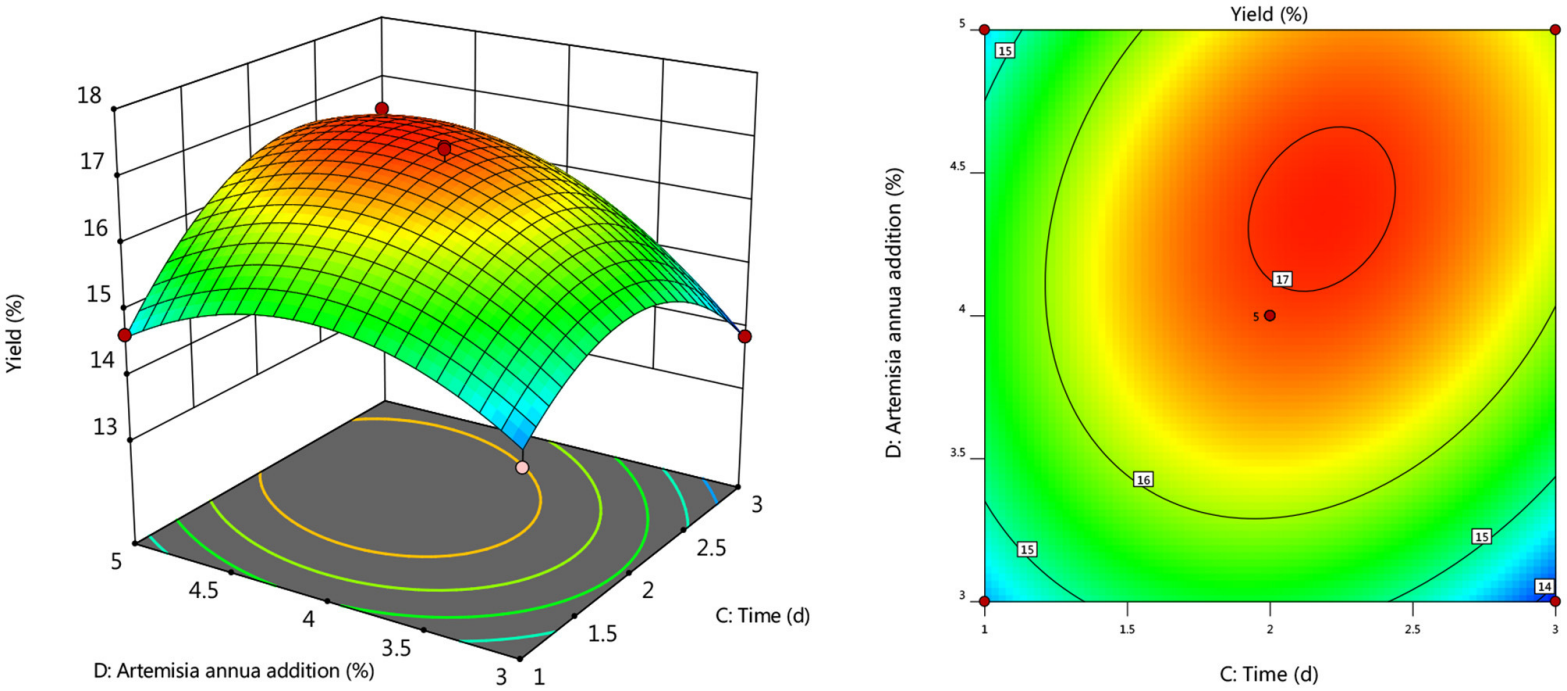
Response plot of the interaction between time and *Artemisia annua* addition on yield.

To further investigate the effects of inoculum, temperature, and time on the yield of *Artemisia* polysaccharide, response surface and contour plots were made according to the regression equation, see Figures 6–11. The steeper the curve trend, the stronger the interaction and the greater the effect on the yield of *Artemisia* polysaccharides, and the contour plot visually reflected the significant degree of the interaction between the factors. With the increase of any two variables the yield of *Artemisia* showed an increasing trend, and after the peak of the interaction between the two, the surface decreased. The interaction between time and *Artemisia* addition was the most significant, with the steepest curve and an elliptical contour line.

### Response Surface Results Optimization

After the analysis of the regression model equation, it was obtained that the best process for the fermentation of *Artemisia* polysaccharides by *Aspergillus niger* was 4.882% inoculum, time 2.181 d, temperature 35.98°C, 4.352% *Artemisia* addition, and the yield of *Artemisia* polysaccharides under this condition was 17.087%. Considering the feasibility of practical operation, it was modified and the fermentation process was determined as 5% inoculum, temperature 36°C, time 2 d, shaker speed 180 r/min, and 4% *Artemisia annua* addition. According to the above conditions, the test was repeated three times and the average worth of *Artemisia* polysaccharide extraction was taken as 17.04 %, which was 0.047% from the theoretical value, indicating that the model is credible and the regression equation can be used for the fermentation of *Artemisia* polysaccharide by *Aspergillus niger*.

### Determination of Polysaccharide Content

The standard curve equation is: A = 0.0614c − 0.0034, *R*^2^= 0.9978.

Where *c* is the polysaccharide concentration (μg/ml) and A is the absorbance value. Put A = 0.575 into the equation, and get c = 9.420, so the purity of polysaccharide after purification: 9.420/10 × 100% = 94.20%.

Table 4 shows the yield of polysaccharides extracted by the fermentation method and the yield of polysaccharides extracted by the traditional hot water method. Compared with the traditional hot water method, the fermentation method takes a long time, but requires low temperature and low energy consumption. It is mainly destroyed by extracellular enzymes produced by the fermentation of *Aspergillus niger*. *Artemisia annua* cells promote polysaccharide release.

**Table 4:** Comparison of fermentation method and hot water method.

**Method**	**Time (h)**	**Temperature (**°**C)**	***Artemisia annua* addition (%)**	**Yield (%)**
Fermentation	48	36	4	17.04
Hot-water	4	90	4	9.81

### Analysis of the Results of Antioxidant Experiments

**Figure 12:**
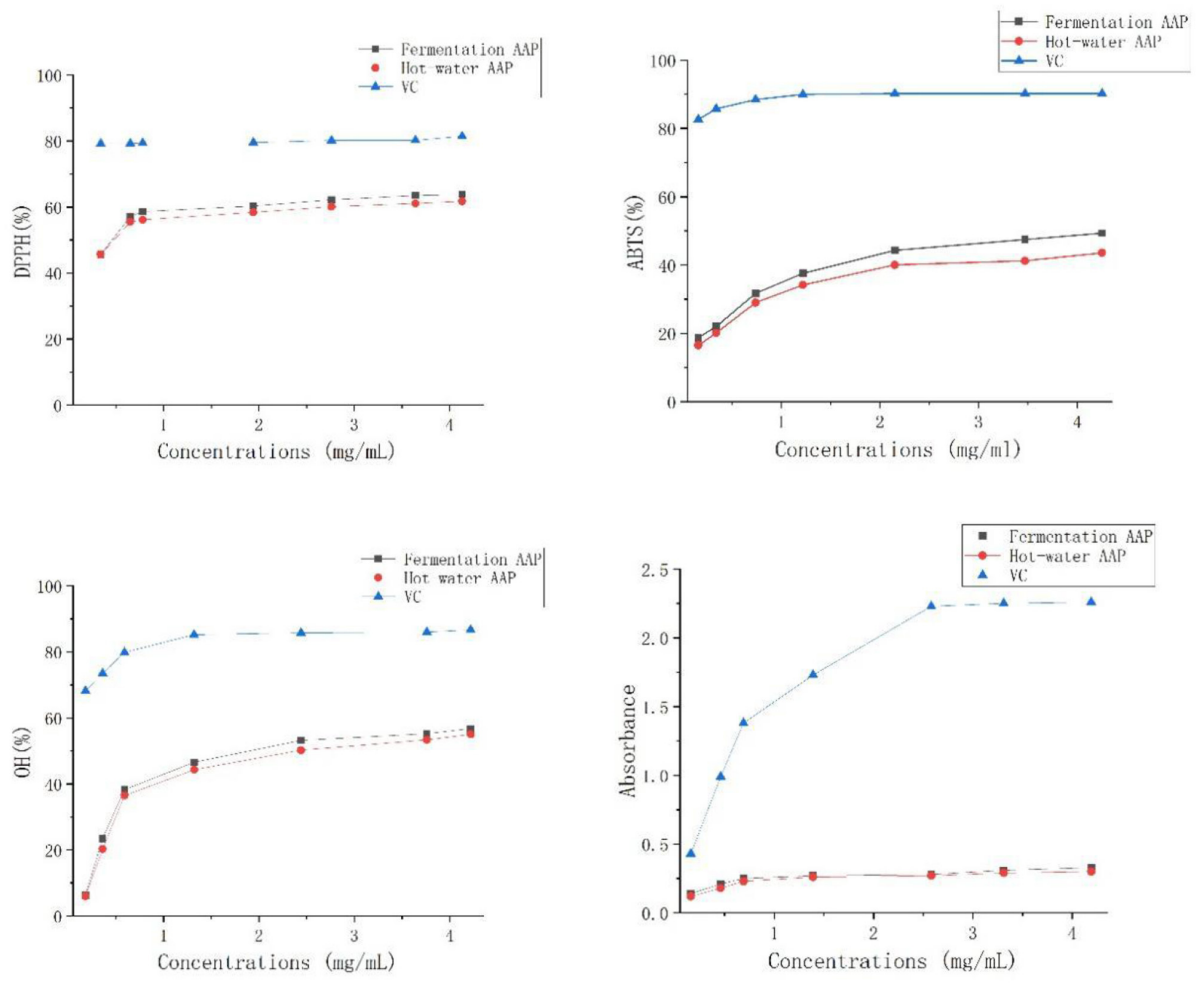
Antioxidant activity assay.

The test was carried out to examine the antioxidant of *Artemisia* polysaccharides using ascorbic acid as a positive control, as shown in Figure 12. *Artemisia* polysaccharides and ascorbic acid showed a scavenging effect on DPPH, ABTS^+^, and OH radicals and there was a dose-dependent relationship. The free radical scavenging rate increased continuously with increasing polysaccharide mass concentration. At the same concentration, the scavenging rate of DPPH, ABTS^+^, and OH radicals by *Artemisia* polysaccharide was weaker than that by ascorbic acid.

## Discussion

Some polysaccharides can participate in cell metabolism for physiological regulation, resulting in a variety of biological functions, such as anti-tumor, anti-virus, anti-oxidation, and immune enhancement, and are widely used as drug delivery carriers because of their non-toxicity and good biocompatibility (29). Deng et al. (30) found that *Artemisia annua* has a high sugar content, but there are not many studies on its extraction.

Li (31) used *Fusarium* rot, *Penicillium chrysogenum*, and *Aspergillus niger* to ferment *Poria*, and found that the water-soluble polysaccharide content of *Poria* fermented by *Aspergillus niger* was the highest, reaching 2.66%, which was 3.86 times that of *Fusarium rot*., and 2.89 times that of *Penicillium flavus*. Gong et al. (32) found that the content of water-soluble tea polysaccharides increased by 5.7 times during the solid-state fermentation process, while polyphenols, flavonoids, catechins, and water-soluble oligosaccharides increased. Sugar content decreased. Using *Aspergillus niger* for fermentation, given the best conditions, the fermentation cycle is short and the growth is vigorous, which can increase the production of enzymes during the fermentation process, thereby destroying the polysaccharide structure of the cell wall (33), and promoting the release of active polysaccharide components, and solid-state fermentation is suitable for filamentous fungi, as it grows in low moisture (34), so *Aspergillus niger* is used for fermentation.

Microbial fermentation can produce enzymes to destroy cell walls, increase active ingredients, reduce toxicity, and regulate the metabolism of reactants to achieve controllable production of products (35). The extraction of polysaccharides by this method is mild and controllable, the operation is simple, and there is no reagent pollution, but the time is relatively long. The ultrasonic extraction method is a physical method for enhanced extraction. The principle is that ultrasonic waves produce a cavitation process in the medium. When the cavitation bubbles collapse, accompanied by high heat, high pressure, and strong shock waves, the cells are ruptured and the active ingredients are released (36). This method extracts polysaccharides. It takes a short time, moderate amount of solvent, produces a high yield, and is a convenient operation, but may lead to the change of polysaccharide structure (37) Zheng et al. (16) extracted polysaccharide from *Artemisia annua* with ultrasonic wave, and the yield was 14.78%. Enzymatic extraction is the use of protease to hydrolyze free protein in plants, resulting in loose plant structure and reduced binding between protein and raw materials, which is beneficial to polysaccharide extraction (38). The cost of this method is slightly higher than that of microbial fermentation, and the enzyme is easily inactivated. With the advantages of mildness, no pollution and good product quality, Shuai et al. (13) used *papain* method to extract polysaccharides from *Artemisia annua* with a yield of nearly 11%. The principle of microwave extraction is to use electromagnetic waves with a frequency between 300 MHz and 300 GHz in plants, causing the internal temperature of the plant to rise, increasing collision and friction to promote cell breakage and exudation of polysaccharides (39). This method is energy-saving, fast, and controllable, but Wei et al. (18) used microwaves to extract polysaccharides from *Artemisia annua*, and the yield was only 9.72%. The traditional hot water extraction method, although the structure is not easily damaged, has high energy consumption, low yield, large amount of extractant, and high cost (40).

Polysaccharide content is an important indicator of microbial fermentation, and the fermentation conditions of microorganisms directly affect the yield. When using *Aspergillus niger* to ferment *Ginkgo biloba*, Wang et al. (34) found that the amount of inoculum did not affect the development process, while the temperature would cause large fluctuations in enzyme activity, sugar content, and bacterial growth. Black spores could be observed after 120 h fermentation at 31°C, 96 h at 31°C and 72 h at 34°C, and secondary germination of *Aspergillus niger* after 96 h fermentation at 34°C, It's a question worth thinking about. Ding et al. (41) used *Aspergillus niger* to ferment *Artemisia annua* and chose to ferment it at 37°C for 5 d, and found that *Aspergillus niger* increased the content of *Artemisinin* and *Artemisinin B* the most among many bacterial groups. The reason can be speculated that *Aspergillus niger* carried out secondary germination, this study also showed that microbial fermentation has a good prospect in the extraction of active ingredients of *Artemisia annua* and provided a theoretical basis. Kamal et al. (42) used banana peel to improve protein production and found that the optimal extraction conditions were 31°C fermentation for 4 d, and the substrate concentration was 19.92%. Therefore, different purposes have different optimal fermentation conditions, and suitable fermentation conditions are very important.

## Conclusions

*Aspergillus niger* can secrete a variety of extracellular enzymes such as lignocellulose hydrolase, acid protease, phytase, glucoamylase, β-glucanase, arabinofuranosidase, and xylanase (43). It is speculated that the extracellular enzymes secreted by *Aspergillus niger* in the fermented *Artemisia annua* have changed its structural properties, thus facilitating the release of polysaccharides. In addition, with the increase of fermentation time and enzyme production, enzymatic reaction occurs, *Artemisia annua* is fully dissolved and cells begin to die or degrade to obtain products. This research adopts fermentation technology, inoculates *Aspergillus niger*, ferments *Artemisia annua* polysaccharide, conducts response surface analysis on the basis of single factor experiment, establishes regression model, and finally determines the optimal fermentation process: *Aspergillus niger* inoculation amount of 5%, temperature of 36°C, the shaking speed was 180 r/min, and the addition of *Artemisia annua* was 4% for 2 d. Under these conditions, the yield of *Artemisia annua* polysaccharide could reach 17.04%, which was not significantly different from the predicted value. The process parameters in this study are highly feasible and the model fits well, which can be used as a reference for the extraction of *Artemisia annua* polysaccharides in the future. According to the analysis of single-factor test and response surface test, the influence degree of each factor is addition amount of *Artemisia annua* > fermentation temperature > inoculation amount of *Aspergillus niger* > fermentation time > shaker rotation speed. Therefore, when using microbial fermentation to extract *Artemisia annua* polysaccharide, In the case of consistent addition of *Artemisia annua*, the temperature factor is given priority, which is beneficial to increase the yield. The yield of *Artemisia annua* polysaccharide extracted by the traditional hot water method was only 9.81%, but its antioxidant activity was almost the same as that of *Artemisia annua* polysaccharide extracted by the fermentation method, and both were weaker than ascorbic acid.

## Data Availability Statement

The original contributions presented in the study are included in the article/supplementary material, further inquiries can be directed to the corresponding author/s .

## Author Contributions

All authors listed have made a substantial, direct, and intellectual contribution to the work and approved it for publication.

## Funding

This work was supported by Academic funding project of Anhui Provincial Department of Education for University Top-notch Talents (No. gxbjZD2021089) and Natural Science Research Foundation of the Department of Education of Anhui Province (Nos. KJ2020A0789 and KJ2021A1166).

## Conflict of Interest

The authors declare that the research was conducted in the absence of any commercial or financial relationships that could be construed as a potential conflict of interest.

## Publisher's Note

All claims expressed in this article are solely those of the authors and do not necessarily represent those of their affiliated organizations, or those of the publisher, the editors and the reviewers. Any product that may be evaluated in this article, or claim that may be made by its manufacturer, is not guaranteed or endorsed by the publisher.
